# Adaptive Block-Based Compressed Video Sensing Based on Saliency Detection and Side Information

**DOI:** 10.3390/e23091184

**Published:** 2021-09-08

**Authors:** Wei Wang, Jianming Wang, Jianhua Chen

**Affiliations:** School of Information Science and Engineering, Yunnan University, Kunming 650000, China; weiwang@mail.ynu.edu.cn (W.W.); yujimmy@163.com (J.W.)

**Keywords:** compressed sensing, side information, saliency detection, fusion sparsity

## Abstract

The setting of the measurement number for each block is very important for a block-based compressed sensing system. However, in practical applications, we only have the initial measurement results of the original signal on the sampling side instead of the original signal itself, therefore, we cannot directly allocate the appropriate measurement number for each block without the sparsity of the original signal. To solve this problem, we propose an adaptive block-based compressed video sensing scheme based on saliency detection and side information. According to the Johnson–Lindenstrauss lemma, we can use the initial measurement results to perform saliency detection and then obtain the saliency value for each block. Meanwhile, a side information frame which is an estimate of the current frame is generated on the reconstruction side by the proposed probability fusion model, and the significant coefficient proportion of each block is estimated through the side information frame. Both the saliency value and significant coefficient proportion can reflect the sparsity of the block. Finally, these two estimates of block sparsity are fused, so that we can simultaneously use intra-frame and inter-frame correlation for block sparsity estimation. Then the measurement number of each block can be allocated according to the fusion sparsity. Besides, we propose a global recovery model based on weighting, which can reduce the block effect of reconstructed frames. The experimental results show that, compared with existing schemes, the proposed scheme can achieve a significant improvement in peak signal-to-noise ratio (PSNR) at the same sampling rate.

## 1. Introduction

Image and video information contains a lot of redundant information, which makes signal compression not only necessary but also feasible. However, this part of the compressible redundant information will be ignored in the traditional video and image acquisition process. This is because the traditional video and image compression algorithms are based on the quantized digital signal, which means that we must first sample and digitize the signal according to the Nyquist sampling theorem, and then compress the digitized signal. That is to say, we first collect the redundant information, and then remove the redundant information collected on the sampling side, in this way, this “remedial” process wastes valuable resources. Compressed sensing (CS) is an innovative signal sampling theory that goes against the conventional wisdom (Nyquist sampling) in signal acquisition [1]. It can unify the signal sampling and compression process into a single step, which means that sampling includes compression. Therefore, it implies that CS is a sub-Nyquist sampling theory. Under the condition where the signal is sparse, CS can directly obtain the compressed form of the signal. In video or image acquisition devices, CS can help combat the hardware limitation, where only a few sensors can be used to achieve higher imaging accuracy (e.g., a single-pixel imaging system) [2,3]. This solves the problem when in practical applications, engineers need to increase the number of pixels integrated on hardware devices as much as possible, while they have to make great efforts to design complex algorithms to compress the collected pixels. It can be said that CS makes the process of information processing more concise and efficient [2]. Therefore, one of the advantages of CS is the ability to image quickly, which is conducive to capture fast moving objects and improve the time resolution of low frame rate cameras to some extent. Moreover, compared with the traditional video coding scheme, CS is more robust against errors, because each measurement result contains all the information of the original signal and is equally important. Therefore, the loss of only a few measurement results in the process of data transmission will not have a great impact on the final reconstruction accuracy. In the practical process of image or video signal processing and communication, especially in the communication process of wireless devices, the resources of the sampling side or encoding side (i.e., the uplink) are more limited, while the resources of the downlink are relatively sufficient. Therefore, it makes CS very suitable for applications, such as wireless sensor networks and wireless video communication devices.

In the process of video/image communication, rate-distortion performance is a very important evaluation index of communication quality. In the compressed video sensing scheme, one of the key factors affecting the rate-distortion performance in video transmission is the setting of the sampling rate. CS theory points out that the minimum sampling rate of sparse signals is determined by the sparsity of the signals. However, in the practical compressed video sensing process, we cannot obtain the original signal instead of the initial measurement results of the signal. Therefore, how to set the appropriate CS sampling rate is a challenging task. Liu et al. [4] proposed a framework to classify blocks into different types depending on their inter-frame correlation, and the sampling and reconstruction strategies are adjusted according to the type of blocks. The measurement number of the static blocks can be predicted indirectly from the corresponding position of the previous frame rather than calculating the measurement number independently, thus improving the efficiency of data acquisition. However, for large-change blocks, the number of measurements is not obtained based on the inter-frame correlation, but based on the texture complexity of the current block itself. A block-based adaptive framework for compressed video sensing was proposed in [5] in which each block in the current frame is adaptively sampled at a rate that depends on the texture complexity and visual saliency [6] of the block. Moreover, for each frame, there will be a different total sampling rate, which is obtained according to the complexity of the frame. In [7], an adaptive compressed video sensing scheme for surveillance video was proposed. In this scheme, researchers used cross validation to compute the number of required measurements. Given a reconstructed foreground, researchers used extra cross-validation measurement results to estimate the area of the true foreground. Then, assuming that the foreground regions of the two consecutive frames are the same, the precalculated phase diagram of the sensing matrix gives the number of measurements for the next frame. The scheme shows good experimental results in the application of surveillance video sequences. An adaptive video CS method based on spatial–temporal difference saliency in the compressed domain was raised in [8], which is convenient for implementation in real-time and holds a higher peak signal-to-noise ratio (PSNR) than non-adaptive methods. In [9], researchers proposed a method that uses a complementary sensor to obtain a low-resolution image, and uses the pulsed cosine transform to extract the saliency information of the image. Then, more CS measurements are allocated to salient blocks but fewer to non-salient blocks, so as to achieve adaptive CS. However, the existence of low-resolution imaging before compressive imaging, affects the advantage of the CS that achieves sub-Nyquist signal sampling. In [10], Zhang et al. proposed an adaptive CS rate assignment method that is based on the standard deviations of image blocks. The experimental results show that the proposed method can effectively improve the quality of reconstructed images. In [11], researchers proposed a saliency-based adaptive CS scheme that allocates more measurements to salient blocks but fewer to non-salient blocks, which extracts the information of saliency by using the difference between CS measurement results. Thus, it avoids the need to obtain the original image in the imaging system.

Assuming that different frames have different frame complexity, each frame may require a different total measurement number. Therefore, before allocating the measurement number of each block in the frame, we first allocate the total measurement number of each frame according to the complexity of each frame. After obtaining the total measurement number of each frame, we can use the measurement results to estimate the block saliency in the measurement domain. The saliency of the frame can reflect the sensitivity degree for human eyes, but it cannot reflect the sparsity of the frame completely. In other words, there is still a lot of room for improvement in the scheme of block sparsity estimation based only on saliency detection. If we have the pixel domain information of the current frame on the sampling side, we can directly estimate the block sparsity according to the proportion of the significant coefficients of each block in the frequency domain, unfortunately we cannot directly obtain the pixel domain information of the current frame. In order to address this thorny issue, we consider making use of the inter-frame correlation of the video sequence to generate the approximate version of the current frame to be sampled on the reconstruction side, namely the side information frame (SIF). Then, the proportion of the significant coefficients of each block calculated by the SIF is fed back to the sampling side, and it is combined with the saliency value calculated by the scheme based on saliency detection to obtain the fusion sparsity for each block. According to the fusion sparsity, we can adaptively allocate the measurement number for each block. In this way, the intra-frame correlation in the measurement domain and the inter-frame correlation in the pixel domain can be used to allocate the measurement number of each block at the same time. Furthermore, the proposed scheme also solves the problems that the pixel domain information of the current frame to be sampled cannot be fully utilized on the sampling side in [4]; and the adaptive sampling in [5] is directly based on pixel domain information, without considering both pixel domain information and measurement domain information.

On the basis of the above consideration, we propose an adaptive block-based compressed video sensing (ABCVS) scheme to comprehensively estimate the measurement number of each block by using the saliency detection in the measurement domain and the side information [12,13] generated on the reconstruction side. Firstly, on the sampling side, the saliency value of each block of the current frame is estimated in the measurement domain. Secondly, on the reconstruction side, the SIF of the current frame is obtained by using previously reconstructed frames to perform the extrapolation based on the proposed probability fusion method. The frequency domain information of the SIF is obtained by performing a discrete cosine transform (DCT), and then the proportion of significant coefficients based on the SIF can be estimated. Finally, the saliency value and the proportion of significant coefficients are weighted and averaged to obtain the fusion sparsity of each block. Then the measurement number of each block is allocated adaptively according to the fusion sparsity. The proposed method can not only solve the inaccuracy of measurement number allocation caused by the mere use of saliency detection in the measurement domain, but also make good use of the inter-frame correlation in video sequences. Considering that the block-based compressed sensing scheme will lead to the block effect of the reconstructed frames, we propose a global recovery model based on weighting to reduce the block effect, so as to further improve the quality of the reconstructed frames.

The rest of this paper is organized as follows. In Section 2, we provide a brief overview of block-based CS (BCS). The proposed ABCVS scheme is presented in Section 3. Extensive simulation results are reported in Section 4 and Section 5 concludes the paper.

## 2. Compressed Sensing Overview

Compressed sensing is a new signal sampling theory. Because a small number of random measurement results from a sparse signal contain enough information to reconstruct the signal, CS can replace traditional signal acquisition methods. In this part, we briefly review the BCS theory. Assume that the current input frame is ***H***, in order to apply BCS, we first divide the current frame ***H*** into *L* nonoverlapping blocks $B_{i}$ (i = 1, 2, …, *L*) with a size of *B* × *B*, then each block is sampled separately. Let $h_{i}$ be the vectorized signal of the i-th block, which is obtained through raster scanning. The corresponding output CS vector, $y_{i}$, (of length *M*) is obtained as follows:

(1) $$y_{i}=\Phi_{Bi}h_{i}$$

where $\Phi_{Bi}\in {\mathbb{R}}^{M_{i}\times B^{2}}$ ($M_{i}\ll B^{2}$) is referred to as the measurement matrix ($\Phi_{Bi}={\left[\varphi_{1}\varphi_{2}\ldots \varphi_{M}\right]}^{T}$) for block $B_{i}$, which is an orthonormalized i.i.d Gaussian matrix [1]. Assume that the signal $h_{j}$ is sparse in the $\Psi_{i}$ domain ($\Psi_{i}={\left[\psi_{1}\psi_{2}\ldots \psi_{B^{2}}\right]}^{T}$ is the transform matrix or sparse basis), we have $h_{i}=\Psi_{i}x_{i}$, where $x_{i}$ is the transform coefficient vector of the signal. Then, (1) can be further written in terms of $x_{i}$ as:

(2) $$y_{i}=\Phi_{Bi}\Psi_{i}x_{i}=A_{Bi}x_{i}\;$$

in which $A_{Bi}=\Phi_{Bi}\Psi_{i}$. CS theory puts forward several general conditions under which the above statement is valid. Firstly, each signal $h_{i}$ to be sampled should be sparse in transform domain $\Psi_{i}$. Frankly speaking, there are only a few nonzero or large elements in the vector $x_{i}$, while many components have zero or much smaller amplitudes, which means that the signal in the vector is sparse. Secondly, the measurement matrix $\Phi_{Bi}$ should show the restricted isometry property (RIP) as follows:

(3) $$\left(1-\delta_{s}\right)∥h_{i}{∥}_{2}^{2}\leq \;∥\Phi_{Bi}h_{i}{∥}_{2}^{2}\;\leq \left(1+\delta_{s}\right)∥h_{i}{∥}_{2}^{2}$$

where $\delta_{s}\in \left(0,1\right)$ is a constant, S denotes the sparse order. Specifically speaking, signal vectors having no more than S nonzero components are said to be S-sparse. Only when the measurement matrix satisfies RIP, can the necessary information needed to reconstruct the original signal be obtained from the measurements. Finally, a stable reconstruction algorithm, with low computational complexity and less requirement for the number of measurements, is also one of the most important components of a CS system. With the measurement vector $y_{i}$ of the i-th block, we can build the following recovery model based on the minimum $\ell^{2}$ and $\ell^{1}$ norms:

(4) $${\tilde{h}}_{i}=\arg\underset{h_{i}}{\min}\left\{{\left|\left|y_{i}-\Phi_{Bi}h_{i}\right|\right|}_{2}^{2}+\lambda {\left|\left|\Psi_{i}h_{i}\right|\right|}_{1}\right\}$$

where ${\left|\left|\cdot \right|\right|}_{2}$ and ${\left|\left|\cdot \right|\right|}_{1}$ are $\ell^{2}$ and $\ell^{1}$ norms respectively, and λ is a fixed regularization factor. Considering that the reconstruction model is a convex optimization problem, the Gradient Projection for Sparse Reconstruction (GPSR) algorithm [14] can be used to solve it.

Combined with the above analysis and the derivations in [1,15,16,17], for a fixed constant *C*, the measurement number $M_{i}$ satisfies the following equation:

(5) $$M_{i}\geq CS\log B^{2}$$

According to (5), we see that an appropriate number of measurements can be determined from the sparse order S, which means the region with higher signal complexity requires more measurement numbers. Thus, when using the same number of measurements for the whole signal, the adaptive block-based compressed sensing (ABCS) scheme can better reconstruct the complex regions of the signal than the traditional non-adaptive BCS.

## 3. The Proposed Scheme

In the block-based compressed video sensing system, different blocks of each frame will have different textures, that is, different blocks will have different sparsity, and the complexity of each frame also varies. Therefore, how to allocate the total measurement number of each frame and the corresponding measurement number of each block appropriately without the pixel domain information of the original video sequence is a challenging task.

**Figure 1:**
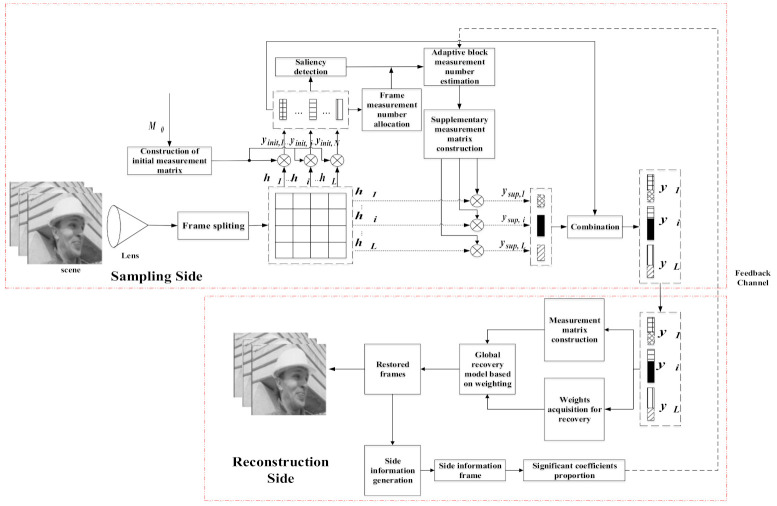
The architecture of ABCVS.

In order to solve this problem, we propose an ABCVS scheme based on saliency detection and side information. The architecture of the proposed scheme is presented in Figure 1. On the sampling side, the lens projects the scene in the field of view into the imaging system and divides the projected image into L blocks with a size of *B × B*. We first construct an initial measurement matrix $\Phi_{init}$ for each block, in which, the fixed measurement number is $M_{0}$. Then we can get the initial measurements $y_{init,i}$ for the i-th block through (2). Once we have the measurements of each block in each frame, we can calculate its own $\ell^{1}$ norm according to the measurements of each block. Based on the $\ell^{1}$ norm of the measurements of each block, we can calculate the measurement domain variance of each frame. Variance can represent the degree of data deviation from the average, which reflects the fluctuation of the data itself, so it can also reflect the complexity of the frame to a certain extent. Based on the complexity difference of each frame in each group of pictures (GOP), the total measurement number of each frame can be assigned. Then, according to the initial measurement results of all the blocks in a frame, we can perform saliency detection by using these initial measurement results to obtain the block saliency $w_{i,saliency}$ of the i-th block. Nonetheless, saliency can only reflect that a block is of interest to human eyes in the video frames, but cannot fully reflect the sparsity of the block, which is the a priori information that the adaptive compressed video sensing system needs to obtain. Therefore, we propose to use both saliency and SIF to estimate the sparsity for each block. Specifically, on the reconstruction side, we use the reconstructed video frames to perform extrapolation based on the proposed probability fusion model (PFM) to obtain the SIF of the current frame to be sampled. Then, the sparsity is determined directly according to the proportion of significant DCT coefficients of the block. It should be noted that we need to use a feedback channel to transmit the proportion of significant coefficients to the sampling side, although the amount of data is very small. In order to make more reasonable and comprehensive use of $w_{i,saliency}$ and SIF, they are fused to get the fusion sparsity $P_{i,fusion}$ for the i-th block according to the summation of the absolute difference (SAD) between frames in the measurement domain on the sampling side. Eventually, we can obtain the measurement number $M_{i,F_{t}}$ of the i-th block in frame $F_{t}$ according to fusion sparsity $P_{i,fusion}$. Meanwhile, the supplementary measurement number $M_{i,F_{t}}-M_{0}$ can be obtained. Through the above steps, we can obtain the supplementary measurement matrix $\Phi_{sup,i}$ for each block, and then obtain the supplementary measurement $y_{sup,i}$ for each block.

In the sequel, we will describe the details of each part.

### 3.1. Frame Measurement Number Allocation

As mentioned earlier, we need to allocate the total measurement number of each frame according to the complexity of each frame. Nonetheless, we cannot get the pixel domain information of the current frame on the sampling side, so it is not easy to calculate the complexity of the current frame. 

In this paper, a frame complexity calculation method based on the measurement domain variance is proposed. Specifically, after the initial sampling, we can obtain the initial measurement vector of each block and calculate the $\ell^{1}$ norm of each initial measurement vector. Based on the $\ell^{1}$ norms of the initial measurement vectors of the blocks, we can use them as sample data to calculate the measurement domain variance of each frame. Variance can measure the deviation of each sample data from the average. That is, the greater the variance, the greater the volatility of the data, and vice versa. Therefore, it can also represent the intra-frame complexity to some extent. Besides, RIP implies that the distance between sparse signals can be well preserved in the measurement domain. From the analysis of the above two aspects, the difference of the measurement domain variance of each frame in each GOP can approximately reflect the complexity difference of each frame in the original pixel domain. Therefore, under a fixed size GOP, the allocation of the total measurement number for the current frame $F_{t}$ can be obtained by:

(6) $$M_{F_{t}}=\;\mathrm{rnd}[\frac{Var_{t}}{\sum_{j=1}^{G}Var_{j}}\cdot \left(G\cdot \left(R_{T}\cdot N_{T}-L\cdot M_{0}\right)\right)+L\cdot M_{0}]$$

in which the function “rnd” rounds its input quantity to the nearest integer. $M_{F_{t}}$ is the total number of measurements for the current frame $F_{t}$, $Var_{t}$ is the measurement domain variance of the current frame $F_{t}$, G is the size of GOP and $N_{T}$ is the total pixel number of each GOP. $R_{T}$ is the total sampling rate for each GOP, that is, the ratio of the total measurement number to the total pixel number in a GOP.

### 3.2. Saliency Detection

Saliency is a method that can reveal the visual characteristic of human perception. Visual saliency can be described by the statistical correlation of visual space, that is, a position with low spatial correlation with the surroundings is salient. In other words, saliency can reflect the sparsity of the signal to some extent. Therefore, we can allocate the measurement number of each block in the current frame based on the saliency information in the ABCVS system. A saliency detection method in [18] is proposed to compute a spatial saliency map by using the luminance contrast between image pixels. Thus, the saliency value of pixel $p\left(k\right)$ in image p is defined as:

(7) $$\xi_{k}=\xi \left[p\left(k\right)\right]=\sum_{j=1}^{N}{\left[p\left(k\right)-p\left(j\right)\right]}^{2}\;$$

where *N* is the total number of pixels in image p. According to (7), we can easily deduce that the saliency of i-th block $B_{i}$ can be calculated by the following equation:

(8) $$\xi_{B_{i}}=\xi_{B}\left[B_{i}\right]=\sum_{j=1}^{L}{\left|\left|B_{i}-B_{j}\right|\right|}_{2}^{2}$$

This method can be used to perform saliency detection in O (N) time order with a low cost and complexity, which is consistent with the low computation complexity of the CS sampling side. However, in a practical CS system, we cannot get the actual digitized pixel information of the current frame to be sampled, which means we cannot estimate the saliency in the pixel domain on the sampling side. Consider that the measurement results in the CS system are obtained by performing dimensional reduction projection of the original signal, which is similar to the convolution step in neural networks. This means that although the overall dimensionality of every CS domain signal is reduced, there is still redundancy between the signals. Therefore, combined with the above and the Johnson–Lindenstrauss lemma [19], we have the following corollary: For an original input signal, if the original form of the signal can be directly used for saliency detection, then the measurement domain signal obtained from the reduced-dimensional projection through the purely random matrix (e.g., Gaussian random matrix), theoretically, is still equivalent to the original signal for saliency detection. Therefore, in the CS system, the saliency of each block can also be calculated by using the initial measurement results on the sampling side, as follows:

(9) $$w_{i,saliency}=w_{saliency}\left[y_{init,i}\right]=\sum_{j=1}^{L}{\left|\left|y_{init,i}-y_{init,j}\right|\right|}_{2}^{2}=\sum_{j=1}^{L}{\left|\left|\Phi_{init}h_{i}-\Phi_{init}h_{j}\right|\right|}_{2}^{2}$$

where $h_{i}$ is the vectorized original signal of the i-th block, and $y_{init,i}$ is the corresponding initial measurement result computed by $\Phi_{init}h_{i}$. In order to facilitate the follow-up processing, we normalize $w_{i,saliency}$ to get $w_{i,saliency}^{*}$.

### 3.3. Side Information Generation

If we have the pixel domain information of the frame to be sampled on the sampling side, then the measurement number allocation will become very direct. Considering that there is a strong inter-frame correlation between video sequence frames, we can perform extrapolation according to the previous reconstructed video frames to obtain the approximate version of the current frame, that is, side information. In order to obtain an accurate SIF, we propose a SIF generation scheme based on PFM, which can fuse two different SIF according to the motion intensity.

**Figure 2:**
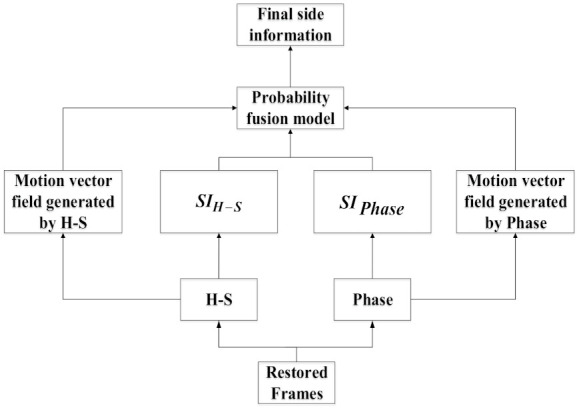
The architecture of side information generation.

The SIF is an approximate version of the current frame to be sampled. According to the proportion of the significant DCT coefficients of the generated SIF, we can directly allocate the number of measurements $M_{i,SI}$ for the i-th block in the frame to be sampled. Here, we use a PFM to generate high-quality SIF. As shown in Figure 2, we use the previously reconstructed frames to perform motion estimation to obtain the motion vectors (MV) of current SIF, and then we can use these MV for motion compensation to obtain the SIF [13]. Specifically, we use the H-S optical flow method [20] and the Phase-based optical flow method [21] (The code is available at https://github.com/owang/PhaseBasedInterpolation accessed on 2 September 2021) to generate the side information $SI_{H-S}$, $SI_{phase}$, respectively. In order to further improve the accuracy of the generated SIF, here, we consider fusing the two generated SIF. The side information $SI_{H-S}$ and $SI_{phase}$ generated by the two methods are divided into nonoverlapping blocks with a size of 8 × 8, respectively. Assuming $\tau_{H-S}$ and $\tau_{Phase}$ are any pair of blocks of side information $SI_{H-S}$ and $SI_{phase}$. The fusion result can be regarded as the weighted average of $\tau_{H-S}$ and $\tau_{Phase}$, which can be represented by the following equation:

(10) $$f\left(\tau_{H-S},\;\tau_{Phase}\right)=\gamma_{H-S}\tau_{H-S}+\gamma_{Phase}\tau_{Phase}$$

where $\gamma_{H-S}$ and $\gamma_{Phase}$ are the weights of $\tau_{H-S}$ and $\tau_{Phase}$, respectively. According to the Bayesian rule we can get:

(11) $$\gamma_{H-S}=p(H-S|f\left(\tau_{H-S},\;\tau_{Phase}\right))$$

(12) $$\;\gamma_{Phase}=p(Phase|f\left(\tau_{H-S},\;\tau_{Phase}\right))$$

The a posteriori probability can be calculated as follows:

(13) $$p\left(H-S|f\left(\tau_{H-S},\;\tau_{Phase}\right)\right)=\frac{p(f\left(\tau_{H-S},\;\tau_{Phase}\right)|H-S)p\left(H-S\right)}{p(f\left(\tau_{H-S},\;\tau_{Phase}\right)|H-S)p\left(H-S\right)+p(f\left(\tau_{H-S},\;\tau_{Phase}\right)|Phase)p\left(Phase\right)}$$

(14) $$p\left(Phase|f\left(\tau_{H-S},\;\tau_{Phase}\right)\right)=\frac{p(f\left(\tau_{H-S},\;\tau_{Phase}\right)|Phase)p\left(\mathrm{Phase}\right)}{p(f\left(\tau_{H-S},\;\tau_{Phase}\right)|H-S)p\left(H-S\right)+p(f\left(\tau_{H-S},\;\tau_{Phase}\right)|Phase)p\left(Phase\right)}$$

Here, $p\left(H-S\right)$ and $p\left(\mathrm{Phase}\right)$ are the a priori probability of two different results. Since we treat the two methods equally, $p\left(H-S\right)=p\left(\mathrm{Phase}\right)=1/2$. The a posteriori probability $p(f\left(\tau_{H-S},\;\tau_{Phase}\right)|H-S)$ and $p(f\left(\tau_{H-S},\;\tau_{Phase}\right)|Phase)$ are very important for the calculation of weights ($\gamma_{H-S}$ and $\gamma_{Phase}$). Besides, for the current frame $F_{t}$, according to the reconstructed video frames $F_{t-2}$ and $F_{t-1}$, we can calculate the SAD for the *i*-th corresponding block of the SIF as follows:

(15) $$SAD_{t}^{i}=\sum_{\left(x,\;y\right)\in B_{i}}\left|F_{t-2}\left(x,y\right)-F_{t-1}\left(x,y\right)\right|$$

Therefore, we can obtain the average block SAD: $SAD_{t,mean}$ of current SIF. Take $SAD_{t,mean}$ as the evaluation index, it is easy to find that the a posteriori probability $p(f\left(\tau_{H-S},\;\tau_{Phase}\right)|H-S)$ and $p(f\left(\tau_{H-S},\;\tau_{Phase}\right)|Phase)$ are related to the sum of the $\ell^{2}$ norms of those MV in the block. According to the central limit theorem, we can assume that $p(f\left(\tau_{H-S},\;\tau_{Phase}\right)|H-S)$ and $p(f\left(\tau_{H-S},\;\tau_{Phase}\right)|Phase)$ are Gaussian probability functions. When the SAD of the current block is less than $SAD_{t,mean}$, it means that the motion intensity of the block at this position is small, so the $\ell^{2}$ norms of the MV in the block should also be small. Then, we have the following expressions:

(16) $$p(f\left(\tau_{H-S},\;\tau_{Phase}\right)|H-S)=p\left(\delta_{H-S}\right)\propto \exp\left(-\delta_{H-S}{}^{2}\right)$$

(17) $$p(f\left(\tau_{H-S},\;\tau_{Phase}\right)|Phase)=p\left(\delta_{Phase}\right)\propto \exp\left(-\delta_{Phase}{}^{2}\right)$$

where, $\delta_{H-S}$ and $\delta_{Phase}$ are the sum of $\ell^{2}$-norms of block MV generated by the H-S optical flow method and Phase-based optical flow method. Let us substitute (13) and (14) with (16) and (17), considering (11) and (12), then we can get the following:

(18) $$\gamma_{H-S}=p\left(H-S|f\left(\tau_{H-S},\;\tau_{Phase}\right)\right)=\frac{\exp\left(\frac{-\delta_{H-S}{}^{2}}{2\sigma_{w}{}^{2}}\right)}{\exp\left(\frac{-\delta_{H-S}{}^{2}}{2\sigma_{w}{}^{2}}\right)+\exp\left(\frac{-\delta_{Phase}{}^{2}}{2\sigma_{w}{}^{2}}\right)}$$

(19) $$\gamma_{Phase}=p(Phase|f\left(\tau_{H-S},\;\tau_{Phase}\right))=\frac{\exp\left(\frac{-\delta_{Phase}{}^{2}}{2\sigma_{w}{}^{2}}\right)}{\exp\left(\frac{-\delta_{H-S}{}^{2}}{2\sigma_{w}{}^{2}}\right)+\exp\left(\frac{-\delta_{Phase}{}^{2}}{2\sigma_{w}{}^{2}}\right)}$$

where $\sigma_{w}{}^{2}$ can adjust the shape of the Gaussian probability function, we empirically set it to 60. When the current block SAD is greater than $SAD_{t,mean}$, it means that the motion intensity of the block at this position is large, so the MV of the block should also be large. According to the above derivation process, we can get the following:

(20) $$\gamma_{H-S}=p\left(H-S|f\left(\tau_{H-S},\;\tau_{Phase}\right)\right)=1-\frac{\exp\left(\frac{-\delta_{H-S}{}^{2}}{2\sigma_{w}{}^{2}}\right)}{\exp\left(\frac{-\delta_{H-S}{}^{2}}{2\sigma_{w}{}^{2}}\right)+\exp\left(\frac{-\delta_{Phase}{}^{2}}{2\sigma_{w}{}^{2}}\right)}$$

(21) $$\gamma_{Phase}=p\left(Phase|f\left(\tau_{H-S},\;\tau_{Phase}\right)\right)=1-\frac{\exp\left(\frac{-\delta_{Phase}{}^{2}}{2\sigma_{w}{}^{2}}\right)}{\exp\left(\frac{-\delta_{H-S}{}^{2}}{2\sigma_{w}{}^{2}}\right)+\exp\left(\frac{-\delta_{Phase}{}^{2}}{2\sigma_{w}{}^{2}}\right)}$$

By performing the above fusion algorithm for each block of the SIF we can eventually get the final side information $SI_{t,final}$.

### 3.4. Adaptive Block Measurement Number Estimation

When the motion intensity of the block in the current frame is small, the accuracy of the corresponding block in the SIF generated by the proposed PFM model is high. Conversely, when the motion intensity of the block in the current frame is large, the accuracy of the corresponding block in the SIF is low. From the above analysis, it is necessary to determine the weighted average of the saliency value and the proportion of the significant coefficients in the SIF according to the motion intensity.

When the final side information $SI_{t,final}$ is generated, the frequency domain information of $SI_{t,final}$ can be obtained by performing DCT. The proportion of significant coefficients in the DCT domain can directly reflect the sparsity of blocks. More specifically, textured blocks usually have many significant coefficients, while smooth blocks usually have relatively few significant coefficients. We generally define the coefficient with an absolute amplitude greater than the threshold value as the significant coefficient, and the threshold value can be obtained as follows:

(22) $$T_{i}=\frac{\sum_{j=1}^{B^{2}}\left|c_{j}\right|}{B^{2}}$$

where $c_{j}$ represents the DCT coefficient of the i-th block. Then, we can get the proportion $\theta_{i,SI}\;$ of the significant coefficients for the i-th block:

(23) $$\theta_{i,SI}=\frac{num_{i,DCT}}{\sum_{j=1}^{L}num_{j,DCT}}$$

where $num_{i,DCT}$ represents the number of significant coefficients of the i-th block. The above $\theta_{i,SI}$ is calculated on the reconstruction side, but in the CS system, the sampling rate is set on the sampling side. Therefore, the feedback channel is needed to feed back the $\theta_{i,SI}$ to the sampling side. However, for the blocks with large motion intensity between video frames, the quality of the generated SIF will be seriously affected by noise. Therefore, the obtained $\theta_{i,SI}$ will also be affected to a certain extent. In order to comprehensively use the information both in the measurement domain and frequency domain to estimate the fusion sparsity $P_{i,fusion}$, we propose the following weighting method, specifically, the SAD calculation in the measurement domain is performed by using the initial measurements of the adjacent frames on the sampling side, which can be defined as follows:

(24) $$SAD_{B_{i}^{t},Mea}=\sum_{j=1}^{M_{0}}\left|B_{i,t}\left(j\right)-B_{i,t-1}\left(j\right)\right|$$

where $B_{i,t}$ represents the vector of measurement results of the i-th block in the frame $F_{t}$ and $SAD_{B_{i}^{t},Mea}$ is the measurement domain SAD of the i-th block in the frame $F_{t}$. Then, we can get the average measurement domain SAD: $T_{SAD,Mea}$ of the current frame $F_{t}$ through the following equation:

(25) $$T_{SAD,Mea}=\frac{\sum_{j=1}^{L}SAD_{B_{i}^{t},Mea}}{L}$$

When $SAD_{B_{i}^{t},Mea}\leq T_{SAD,Mea}$, we can get the weight $\rho_{i,SI}$ of $\theta_{i,SI}$ through the following equation:

(26) $$\rho_{i,SI}=\frac{T_{SAD,Mea}-SAD_{B_{i}^{t},Mea}\;}{T_{SAD,Mea}}$$

Therefore, the weight $\rho_{i,Saliency}$ of $w_{i,saliency}^{*}$ can be obtained through the following equation:

(27) $$\rho_{i,Saliency}=1-\rho_{i,SI}$$

Eventually, the fusion sparsity $P_{i,fusion}$ of the i-th block can be obtained as follows:

(28) $$P_{i,fusion}=\frac{(\rho_{i,Saliency}\cdot \;w_{i,saliency}^{*}+\rho_{i,SI}\cdot \theta_{i,SI})\;}{\sum_{j=1}^{L}(\rho_{j,Saliency}\cdot \;w_{j,saliency}^{*}+\rho_{j,SI}\cdot \theta_{j,SI})\;}$$

When $SAD_{B_{i}^{t},Mea}\geq {\;T}_{SAD,Mea}$, the motion intensity between the video frames is large in the i-th block, which means a low similarity between the generated SIF and the original frame. Therefore, the reference value of $\theta_{i,SI}$ is low. In this case, we set the weight of $\theta_{i,SI}$ to zero, which means $w_{i,saliency}^{*}$ will be completely retained, i.e., the weight of $w_{i,saliency}^{*}$ is one. Then, the fusion sparsity $P_{i,fusion}$ of the i-th block can be obtained as follows:

(29) $$P_{i,fusion}=\frac{w_{i,saliency}^{*}\;}{\sum_{j=1}^{L}(\rho_{j,Saliency}\cdot \;w_{j,saliency}^{*}+\rho_{j,SI}\cdot \theta_{j,SI})\;}$$

Then, the measurement number $M_{i,F_{t}}$ for the i-th block $B_{i}$ in frame $F_{t}$ can be calculated using the fusion sparsity $P_{i,fusion}$ as follows:

(30) $$M_{i,F_{t}}=\;\mathrm{rnd}\left[P_{i,fusion}\cdot \left(M_{F_{t}}-L\cdot M_{0}\right)+M_{0}\right]$$

### 3.5. Global Recovery Model Based on Weighting

When the measurement vector $y_{i}$ is received on the reconstruction side, we can reconstruct each block independently through (4). To solve the above convex optimization problem, many techniques have been proposed in the literature. The gradient projection for sparse reconstruction (GPSR) [14] is one of the most efficient algorithms. However, because the spectrum information is leaked in the process of block-based reconstruction, and the convergence of the recovery algorithm varies rapidly according to the number of measurements of each block, the block recovery model will lead to a serious block effect. Especially for the adaptive rate sampling scheme, the uneven distribution of sampling resources will aggravate the block effect in reconstructed video frames. However, this can be improved by reconstructing the frame using the CS measurement results of all blocks. Specifically, the CS measurement results of all blocks are arranged in columns as follows:

(31) $$y=\left[\begin{array}{c} y_{1}\\ \vdots \\ y_{i}\\ \vdots \\ y_{L} \end{array}\right]=\left[\begin{array}{ccccc} \Phi_{B_{1}} & & & & \\ & \ddots & & & \\ & & \Phi_{B_{i}} & & \\ & & & \ddots & \\ & & & & \Phi_{B_{L}} \end{array}\right]\left[\begin{array}{c} h_{1}\\ \vdots \\ h_{i}\\ \vdots \\ h_{L} \end{array}\right]$$

in which $\Phi_{B_{i}}=\left[\begin{array}{c} \Phi_{init}\\ \Phi_{sup,i} \end{array}\right]$ assume

(32) $$\Phi =\left[\begin{array}{ccccc} \Phi_{B_{1}{}_{}} & & & & \\ & \ddots & & & \\ & & \Phi_{B_{i}} & & \\ & & & \ddots & \\ & & & & \Phi_{B_{L}} \end{array}\right]$$

and then introduce the elementary matrix E to rearrange the column vectors block by block into the raster-scanning column vector of the frame as follows:

(33) $$\left[\begin{array}{c} h_{1}\\ \vdots \\ h_{i}\\ \vdots \\ h_{L} \end{array}\right]=E\cdot h$$

Let us substitute (31) with (32) and (33), and then we can get:

(34) y=Φ·E·h=Θ·h

where Θ=Φ·E. Further, the global recovery model can be constructed as follows:

(35) $$\tilde{h}=\arg\underset{h}{\min}\left\{{\left|\left|y-\Theta \cdot h\right|\right|}_{2}^{2}+\lambda {\left|\left|\Psi h\right|\right|}_{1}\right\}$$

where Ψ is the transform matrix. The above global recovery model can be solved by the GPSR algorithm. Besides, the above model can find the sparse coefficients of h directly in Ψ space, so it effectively solves the problem that the global sparsity of the frame cannot be fully utilized by performing the block reconstruction model (4), thereby effectively suppressing the block effect. However, the global recovery model cannot adjust the reconstruction quality of each block according to the complexity of each block, which leads to the low reconstruction accuracy of some blocks with high complexity. Therefore, we propose a global recovery model based on weighting, which can improve the reconstruction quality of high complexity areas in video frames. Considering that the number of measurements received on the reconstruction side can indirectly reflect the complexity of blocks to a certain degree, and in the natural video sequence, the complexity of the block is determined by many factors. Therefore, according to the central limit theorem, we assume $\left(B^{2}-M_{i,F_{t}}+M_{0}\right)$ is the random variable with the Gaussian distribution. Then, we can have the following weights calculation method:

(36) $$\eta_{i}=\frac{\exp\left(\frac{-{\left(B^{2}-M_{i,F_{t}}+M_{0}\right)}^{2}}{2\sigma_{m}{}^{2}}\right)}{\sum_{j=1}^{L}\exp\left(\frac{-{\left(B^{2}-M_{j,F_{t}}+M_{0}\right)}^{2}}{2\sigma_{m}{}^{2}}\right)}$$

where $\eta_{i}$ is the weight of the proposed global recovery model based on weighting. The parameter $\sigma_{m}{}^{2}$ can be used to adjust the shape of the Gaussian distribution function. Then, we can construct the global recovery model based on weighting as follows:

(37) $$\tilde{h}=\arg\underset{h}{\min}\left\{\overset{L}{\underset{i=1}{\sum }}\eta_{i}^{2}{\left|\left|y_{i}-\Phi_{Bi}h_{i}\right|\right|}_{2}^{2}+\lambda {\left|\left|\Psi h\right|\right|}_{1}\right\}$$

From the proposed model (37), it is easy to find that the larger $\eta_{i}$ is, the closer $\Phi_{Bi}h_{i}$ is to $y_{i}$. Therefore, the proposed model (37) makes the reconstruction results of the blocks with high complexity closer to the original blocks. Next, we construct the following diagonal matrix W:

(38) $$W=\;\mathrm{diag}\;\left[\overset{M_{1}}{\overbrace{\eta_{1}\cdots \eta_{1}}},\cdots ,\overset{M_{i}}{\overbrace{\eta_{i}\cdots \eta_{i}}},\cdots ,\overset{M_{L}}{\overbrace{\eta_{L}\cdots \eta_{L}}}\right]$$

By using the diagonal matrix W, (37) can be represented as follows:

(39) $$\tilde{h}=\arg\underset{h}{\min}\left\{{\left|\left|W\left(y-\Theta \cdot h\right)\right|\right|}_{2}^{2}+\lambda {\left|\left|\Psi h\right|\right|}_{1}\right\}$$

To further simplify the expression of (39) above, we can obtain the following:

(40) $$\tilde{h}=\arg\underset{h}{\min}\left\{||\tilde{y}-\Gamma \cdot h)|{|}_{2}^{2}+\lambda {\left|\left|\Psi h\right|\right|}_{1}\right\}$$

among which $\tilde{y}=Wy$, Γ=WΘ, and λ is the adjustable parameter. It is easy to find that the above model (40) is still the minimum $\ell^{1}$ − $\ell^{2}$ norm model, so it can still be solved by the GPSR algorithm.

## 4. Simulation Results

We tested the performance of the proposed scheme under different experimental conditions. We applied the proposed scheme which is described in the previous section to eight standard video sequences: Foreman, Stephan, football, Bus, Crew, Highway, Table-Tennis, Australia, which were assumed to be the real raster scan videos in our experiments. (Considering that the number of frames of the original standard video sequences were not the same, we took the first 80 frames of each sequence for all experiments.) To begin with, the performances of several submodules were evaluated respectively. Then, we compared the overall performance of the proposed scheme with that of the existing scheme. The schemes that are compared in this paper are: non-adaptive scheme (each block has the same number of measurements), adaptive scheme based on block classification [4] and adaptive scheme based on texture complexity and visual saliency [5]. (We compared the proposed sampling scheme with the adaptive sampling scheme in [4] and [5], and all the reconstruction schemes were solved by GPSR.) For memory reasons, we downsampled each frame of the CIF@30 Hz format video sequences to 256 × 256. (The standard video sequences are available at http://trace.eas.asu.edu/yuv/index.html and http://amalia.img.lx.it.pt/~tgsb/H264_test/ accessed on 2 September 2021) In each part of the experiment, the block size B was set to 16 and the total sampling rate $R_{T}$ was set between 0.3 and 0.5 in the overall performance comparison experiment. The sparse basis Ψ was a Daubechies orthogonal wavelet of length 4 and the initial measurement number $M_{0}$ of each block was set to be rnd[$0.3\cdot R_{T}\cdot B^{2}$].

### 4.1. Evaluation of Different GOP Sizes

**Figure 3:**
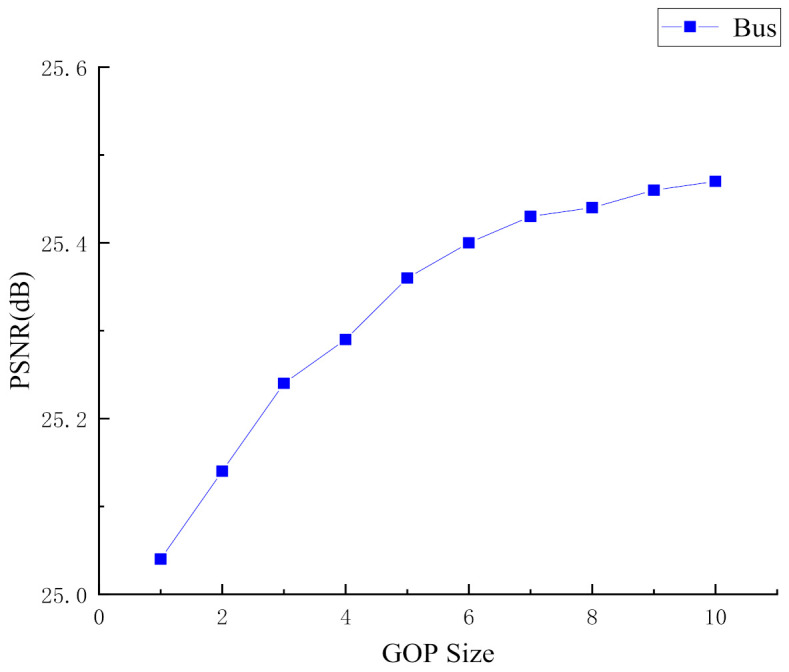
Influence of GOP size on the performance of the proposed scheme (Bus sequence, sampling rate = 0.3).

In our ABCVS architecture, we divide the video sequence by GOP size. The total number of measurements of each GOP is allocated to each frame of the current group according to the complexity of the frame, and then the total number of measurements of each frame is adaptively allocated to each block. It can be seen that the setting of the GOP size will affect the allocation of the measurement number to a certain extent, thereby affecting the quality of reconstructed frames. In this section, we evaluate the influence of the GOP size setting on the overall performance of the proposed scheme. We evaluate the performance of the Bus sequence at different GOP sizes when the sampling rate is 0.3. The average PSNR of the reconstructed video frames versus the GOP size is plotted in Figure 3.

It can be seen from Figure 3 that with the increase of the GOP size, the average PSNR of reconstructed video frames will increase accordingly. But the increasing trend will slow down as the size of the GOP increases. Especially when the GOP is greater than 5, the increase of the average PSNR of the reconstructed video frames is very limited. This may be because, when the size of the GOP is greater than 5, the difference of frame complexity in each GOP will no longer increase significantly with the increase of the GOP size, i.e., the allocation of the measurement number of each frame will not change much. As a result, the quality of the reconstructed frames is not obviously improved. Therefore, in order to minimize the storage burden on the sampling side and maintain the fluency of the video sequence, the size of the GOP is set to 5 in our proposed scheme.

### 4.2. Evaluation of Side Information Generation

**Figure 4:**
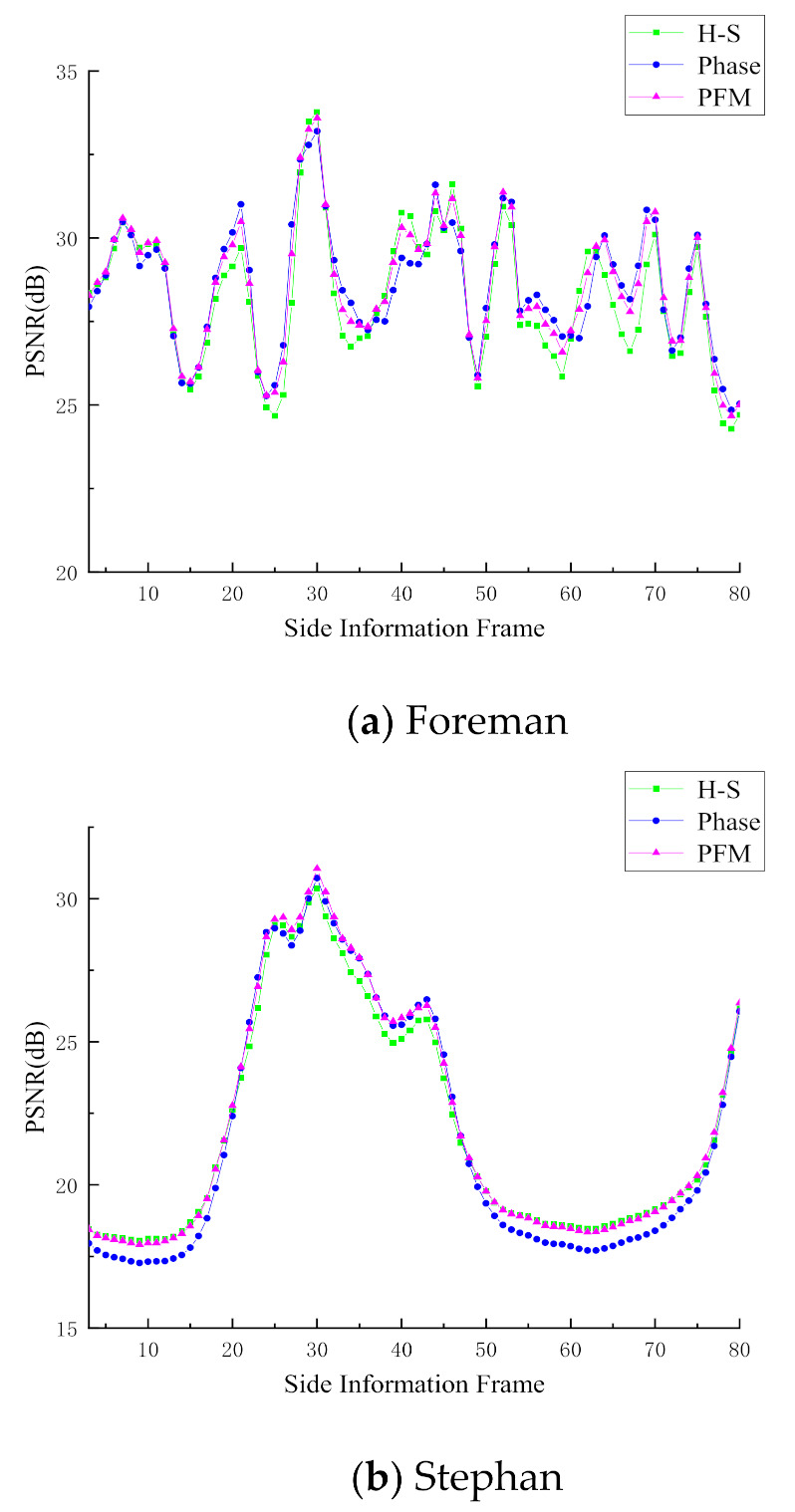

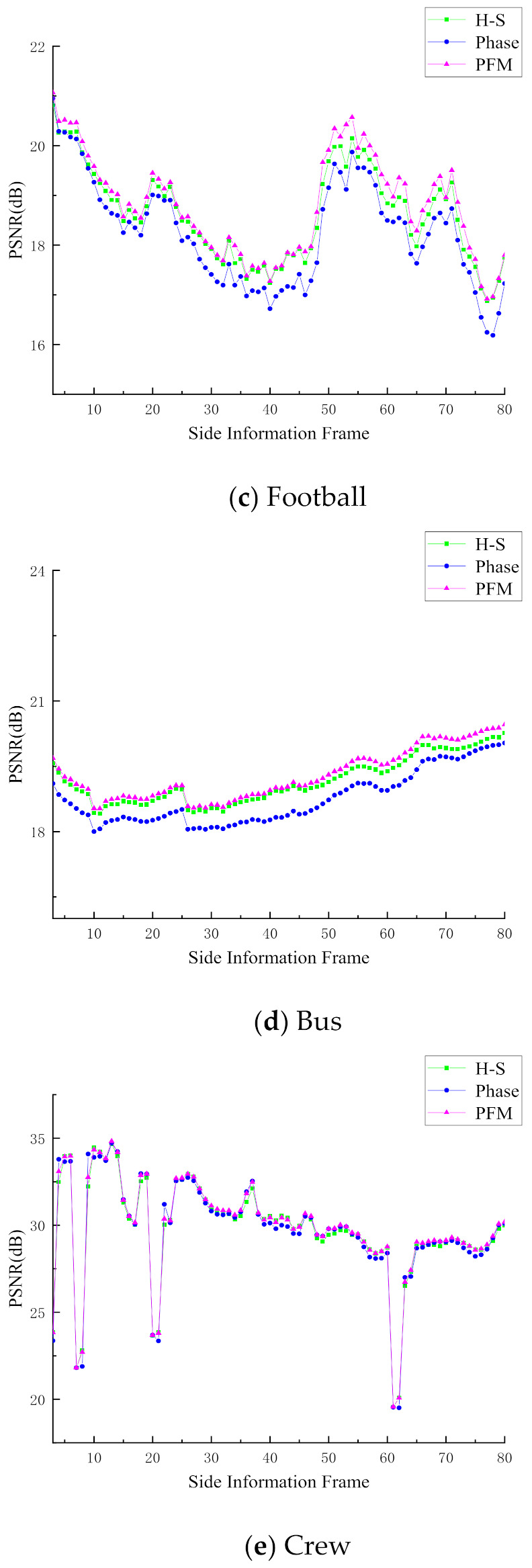

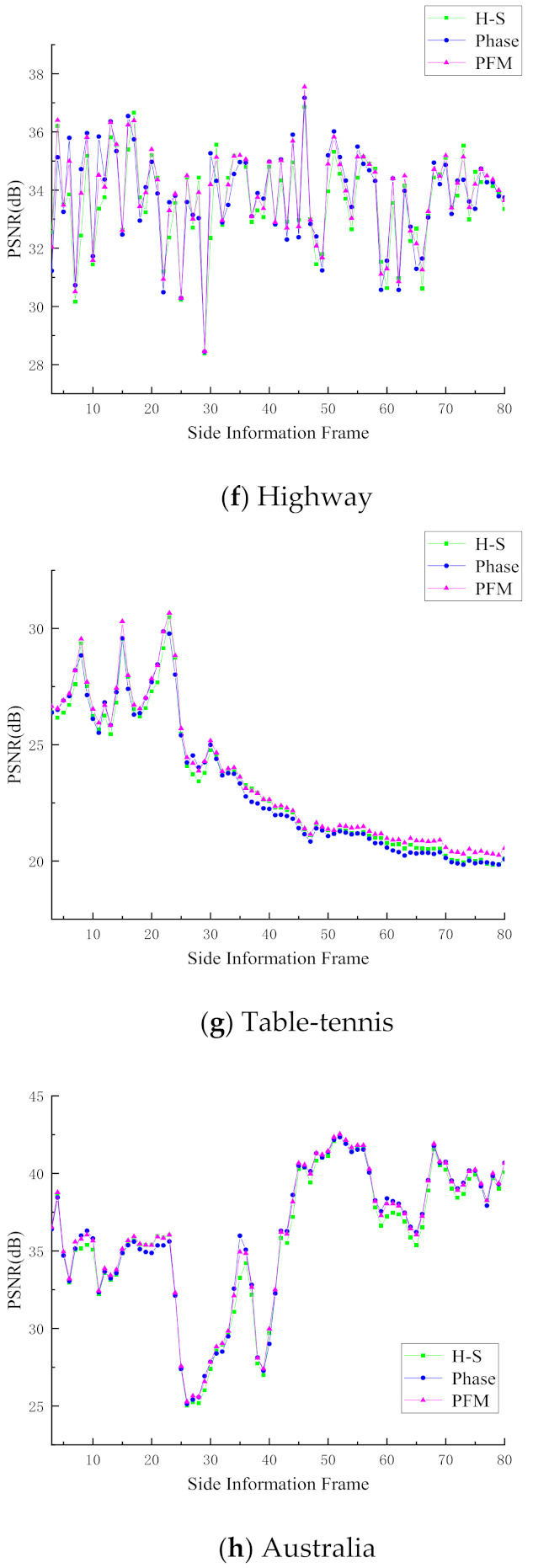
PSNR comparison of SIF generated by different schemes.

In this section, the results of the side information generation module are reported, and the test sequences are as follows: Foreman, Stephan, football, Bus, Crew, Highway, Table-Tennis, Australia. In order to objectively compare the proposed scheme with other schemes, all the extrapolation processes are performed on the original video frames. In the experiment, the pixel accuracy of extrapolation is 1/4 pixel. We use peak-signal-to-noise ratio (PSNR) to evaluate the quality of the SIF generated by various schemes. Figure 4 shows the performance comparison results of various schemes, and the average PSNR results of each scheme are given in Table 1.

**Figure 5:**
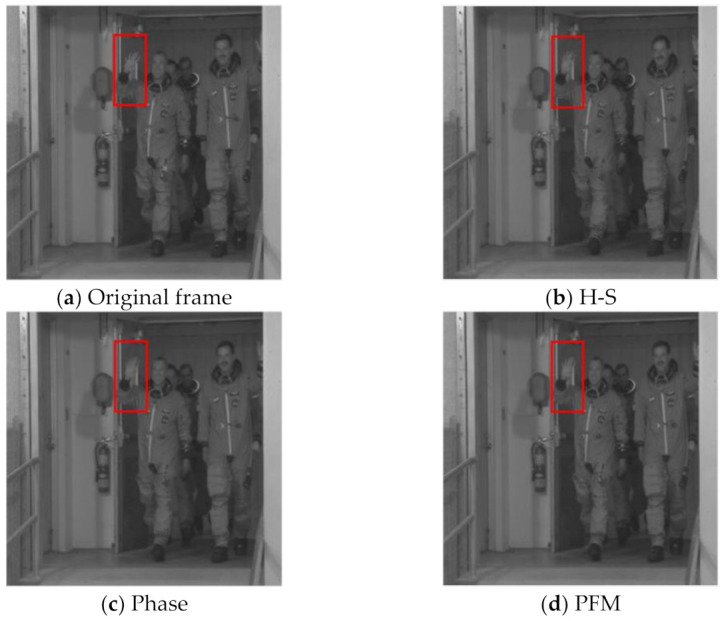
Comparison of subjective quality of the SIF generated by different schemes (The 12th frame of the Crew sequence).

According to Figure 4, we can see that the quality of SIF generated by the proposed scheme based on PFM is better than that of the comparison scheme as a whole. Table 1 reports the average PSNR of SIF. For instance, for the Foreman sequence, the average quality of SIF generated by the proposed PFM is slightly better than that generated by the H-S scheme and the Phase scheme. For the Stephan sequence, the average PSNR of SIF generated by the PFM scheme is 0.23 dB and 0.49 dB higher than that of the H-S scheme and the Phase scheme, respectively. For the Football sequence and the Bus sequence, the proposed PFM scheme shows good performance. Compared with the H-S and Phase schemes, the average PSNR of the generated SIF increases by more than 0.5 dB. This may be due to the large difference between the MV generated by the H-S scheme and the Phase scheme in the Football sequence and the Bus sequence, that is, the MV generated by the H-S scheme are closer to the real situation for some blocks, and the MV generated by the Phase scheme are quite different from the real motion. In turn, the MV generated by the Phase scheme are more accurate for other blocks, while the MV generated by the H-S scheme have large errors. According to the description of the previous section, we know that the proposed PFM is very suitable for SIF improvement in these kinds of situations. For the Crew sequence, the average PSNR of the SIF generated by the proposed PFM scheme is about 0.2 dB higher than that of the H-S scheme and the Phase scheme. For the Highway sequence and the Australia sequence, due to the low motion intensity of the video sequence itself, the accuracy of the MV generated by the H-S scheme and the Phase scheme is high enough. In other words, the difference between the MV generated by the two methods is small and very close to the real MV. Therefore, the improvement effect of the proposed PFM is very limited. For the Table-tennis sequence, the average PSNR of the SIF generated by the proposed PFM scheme is about 0.3 dB higher than that generated by the H-S scheme and the Phase scheme. However, due to the large overall motion intensity of the Table-tennis sequence, the average PSNR of the SIF is not high. Figure 5 shows a comparison of the subjective quality of the generated SIF. It can be found that the subjective quality of the side information generated by the proposed PFM scheme is better than that of the Phase scheme and the H-S scheme. For example, by comparing the parts marked in the red box in Figure 5, it can be found that the clarity of the hand in the SIF generated by the proposed PFM scheme is closest to the original frame. With the SIF, we can use them to calculate the proportion of the significant coefficients of each block. Considering that the SIF are generated on the reconstruction side, we need to use the feedback channel to transmit the obtained proportion of the significant coefficients to the sampling side, in which the feedback channel is a very common channel in the distributed video coding system [12,13]. In this paper, the feedback channel is used to feed back the obtained proportion of each block to the sampling side, that is, each block only needs to feed back a proportion value to the sampling side.

### 4.3. Evaluation of the Global Recovery Model Based on Weighting

**Figure 6:**
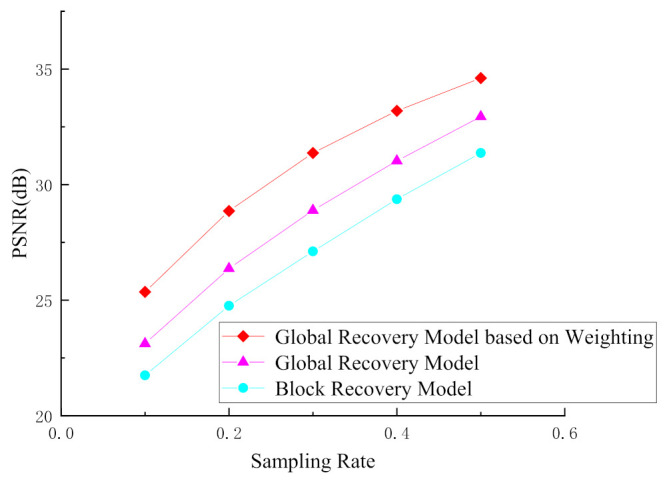
Performance comparison of different recovery models.

**Figure 7:**
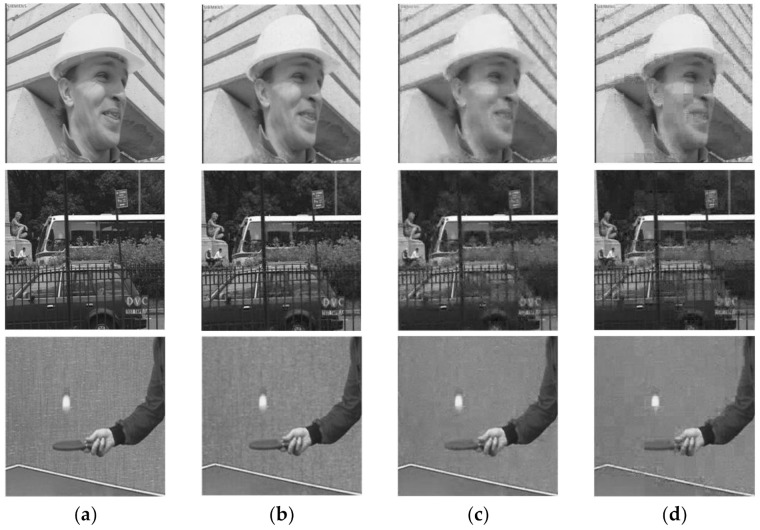
Subjective quality comparison of reconstructed frames recovered by different recovery models. The 10th frame of the Foreman sequence, Bus sequence and Table-tennis sequence, sampling rate = 0.3. (**a**) Original frame, (**b**) Global recovery model based on weighting, (**c**) Global recovery model, (**d**) Block recovery model.

In this section, we verify the performance of the proposed global recovery model based on weighting, in which the parameter $\sigma_{m}{}^{2}$ is set to 40 empirically. Figure 6 shows the comparison of the recovery performance of different recovery models for the Foreman sequence. It is easy to find from Figure 6 that the average PSNR of the reconstructed frames recovered by the global recovery model is increased by more than 1 dB compared with the block recovery model. Moreover, compared with the global recovery model, the global recovery model based on weighting improves the average PSNR of the reconstructed frames by about 2 dB. Figure 7 shows the comparison of the subjective quality of the reconstructed frames recovered by different recovery models. It can be seen from Figure 7 that the video frames restored by the block recovery model have an obvious block effect, while in the video frames reconstructed by the global recovery model, the problem of block effect is completely solved. On the basis of the global recovery model, the global recovery model based on weighting proposed in this paper not only weakens the block effect, but also improves the overall quality of the reconstructed frames, especially the reconstruction quality of some detailed information in the video frame, which has been significantly improved. For example, in Figure 7c, although the global recovery model solves the problem of block effect in the reconstructed frames caused by the block recovery model, using only the global recovery model cannot recover some of the details in the frames. The proposed global recovery model based on weighting is more advantageous in the reconstruction of detailed information. Comparing (a), (b), and (c) in Figure 7, we can see that the texture, the edge of the building, the ping-pong ball and the details of the face in (b) reconstructed using the global recovery model based on weighting, are clearer than those of (c).

### 4.4. Overall Performance Comparison

This section presents the comparison of the simulation results between the proposed compressed video sensing scheme and the other schemes: non-adaptive scheme, [4,5]. (The allocation of the measurement number in [4] mainly depends on the measurement domain information, and the pixel domain information of the current frame to be sampled is not used. The adaptive sampling in [5] is directly based on pixel domain information, but is not combined with measurement domain information to perform a better measurement number allocation.) All the schemes are solved by the GPSR algorithm, in which the regularization factor λ is set to 0.3. Since we need to use the reconstructed frames to perform extrapolation on the reconstruction side to generate SIF, we use a compressed sensing scheme based only on the saliency detection described in Section 3.2 to sample and reconstruct the first and second frames of the scene at the beginning (the sampling rate is set to 0.8 for higher quality). The outperformance of the proposed scheme over the other four, for the Foreman, Stephan, football, Bus, Crew, Highway, Table-Tennis, and Australia sequences, is exhibited in Table 2. It can be found from Table 2 that the proposed scheme is better than the comparison schemes as a whole, because the proposed scheme makes comprehensive use of the intra-frame correlation in the measurement domain and the inter-frame correlation in the pixel domain to allocate the number of measurements. For the Foreman sequences, because the volatility of the video itself is small, i.e., the motion intensity of the video is small, the quality of the generated SIF is higher, which means the reference value of the generated SIF is greater. The allocation of the measurement number for each block is more reasonable. Therefore, the average PSNR of the reconstructed frames of the proposed scheme is better than that of the comparison schemes. In other words, the proposed scheme can feed back the pixel domain information of the current frame to the sampling side through the feedback channel, and can fuse the information of the measurement domain and the pixel domain to perform a more accurate measurement number allocation. When the sampling rate is 0.5, the average PSNR of the reconstructed frames of the proposed scheme is about 1.4 dB higher than that of the non-adaptive, and 0.41 dB, and 0.89 dB higher than that of [4], and [5], respectively. For sports video sequences, such as the Stephan sequence, Football sequence, and Table-tennis sequence, the improvement of the proposed scheme is not obvious. This is because the motion intensity of these video sequences is high, so the accuracy of the SIF generated by extrapolation is low. The low accuracy of the SIF means that the accurate sparsity information that the SIF can provide is limited. In particular, for the Bus sequence, because the background of the whole video is changing and the texture complexity of different regions of the video frames varies greatly, i.e., the sparsity of different regions is very different. In this case, the proposed scheme can give full play to its advantages. When the sampling rate is 0.4, the average PSNR of the reconstructed video frames generated by the proposed scheme is 2.57 dB, 2.01 dB, and 2.29 dB higher than that of non-adaptive scheme, [4], and [5], respectively.

## 5. Conclusions

In this paper, an adaptive block-based compressed video sensing scheme based on saliency detection and side information is proposed. The saliency detection is performed in the measurement domain which can obtain the preliminary sparsity according to the saliency value of each block. The side information frame is generated by the proposed probability fusion method which can fully use the inter-frame correlation to perform a sparsity estimation for each block. Based on the saliency value of each block in the measurement domain and the significant coefficients proportion of each block in the generated side information frame, we can use both intra-frame correlation and inter-frame correlation to estimate the block sparsity, and then adaptively allocate the measurement number for each block to be sampled. On the reconstruction side, we use the global recovery model based on weighting to reconstruct each frame, which can suppress the block effect caused by the block recovery model. The simulation results show that the proposed adaptive block-based video compressed sensing scheme can effectively solve the problem of inaccurate estimation of the measurement number of each block on the sampling side. In other words, the proposed scheme can effectively improve the reconstruction quality of video frames while the total sampling rate is constant.

## Figures and Tables

**Table 1:** The average PSNR of SIF generated by different schemes.

Sequence	H-S [20]	Phase [21]	PFM
Foreman	28.24 dB	28.47 dB	28.55 dB
Stephan	21.95 dB	21.68 dB	22.17 dB
Football	18.62 dB	18.25 dB	18.75 dB
Bus	19.16 dB	18.76 dB	19.30 dB
Crew	29.72 dB	29.69 dB	29.88 dB
Highway	33.60 dB	33.74 dB	33.87 dB
Table-tennis	23.25 dB	23.20 dB	23.56 dB
Australia	35.71 dB	36.01 dB	36.11 dB

**Table 2:** Average PSNR Comparison of Various Schemes with Different Sampling Rates.

Sequence	Method	Sampling Rate		
		0.3	0.4	0.5
Foreman	Non-adaptive	28.74 dB	30.94 dB	33.12 dB
	[4]	29.66 dB	32.00 dB	34.20 dB
	[5]	30.35 dB	32.12 dB	33.72 dB
	Proposed	31.37 dB	33.19 dB	34.61 dB
Stephan	Non-adaptive	22.88 dB	24.50 dB	26.28 dB
	[4]	23.64 dB	25.63 dB	27.78 dB
	[5]	23.55 dB	24.86 dB	26.58 dB
	Proposed	25.99 dB	27.76 dB	29.51 dB
Football	Non-adaptive	26.42 dB	28.42 dB	30.48 dB
	[4]	27.57 dB	28.73 dB	31.12 dB
	[5]	29.45 dB	31.52 dB	33.73 dB
	Proposed	30.08 dB	32.24 dB	34.26 dB
Bus	Non-adaptive	23.43 dB	25.09 dB	26.14 dB
	[4]	23.71 dB	25.65 dB	27.86 dB
	[5]	23.84 dB	25.37 dB	26.43 dB
	Proposed	25.79 dB	27.66 dB	29.24 dB
Crew	Non-adaptive	31.37 dB	33.03 dB	35.49 dB
	[4]	31.49 dB	33.23 dB	35.56 dB
	[5]	31.63 dB	33.26 dB	35.78 dB
	Proposed	33.44 dB	35.47 dB	37.06 dB
Highway	Non-adaptive	33.66 dB	35.55 dB	37.38 dB
	[4]	35.12 dB	37.30 dB	38.87 dB
	[5]	34.12 dB	36.29 dB	37.89 dB
	Proposed	35.21 dB	37.58 dB	39.40 dB
Table-tennis	Non-adaptive	28.38 dB	30.29 dB	32.24 dB
	[4]	30.86 dB	32.80 dB	34.54 dB
	[5]	29.05 dB	30.88 dB	32.76 dB
	Proposed	31.16 dB	33.25 dB	34.93 dB
Australia	Non-adaptive	33.46 dB	34.98 dB	36.24 dB
	[4]	34.28 dB	36.72 dB	37.25 dB
	[5]	33.76 dB	35.78 dB	36.55 dB
	Proposed	34.42 dB	36.92 dB	37.85 dB

## Data Availability

Not Applicable.
